# Endoscopic Versus Microscopic Cartilage Myringoplasty in Chronic Otitis Media

**DOI:** 10.22038/ijorl.2020.44015.2453

**Published:** 2020-09

**Authors:** Ahmad Daneshi, Ali Daneshvar, Alimohamad Asghari, Mohammad Farhadi, Saleh Mohebbi, Mohammad Mohseni, Nasrin Yazdani, Shabahang Mohammadi, Farideh Hosseinzadeh

**Affiliations:** 1 *ENT and Head & Neck Research Center* *, * *The * *Five Senses Health Institute* *, * *Iran University of Medical Sciences, Tehran, Iran.*; 2 *Skull Base * *Research Center* *, * *The * *Five Senses Health Institute* *, * *Iran University of Medical Sciences, Tehran, Iran.*; 3 *Otorhinolaryngology Research Center, Tehran University of Medical Sciences, Amir Alam Hospital, Tehran, Iran.*

**Keywords:** Cartilage, Endoscopic, Myringoplasty, Tympanoplasty

## Abstract

**Introduction:**

Operations on the tympanic membrane of the middle ear, myringoplasty, and tympanoplasty are now widely accepted, and attempts are underway all over the world to standardize the surgical techniques. This study aimed to compare postoperative outcomes of endoscopic and microscopic cartilage myringoplasty in patients suffering from chronic otitis media (COM).

**Materials and Methods:**

This clinical trial study compared 130 patients with COM who underwent transcanal endoscopic myringoplasty by repairing perforation using auricular concha cartilage under general anesthesia (n=75) and conventional repairing method by postauricular incision and tympanomeatal flap elevation under microscopic surgery (n=55).

**Results:**

According to the results, there was no significant difference between the two groups in terms of hearing gain 1, 6, and 12 months after surgery (P=0.063); however, higher hearing gain scores were observed in the endoscopic group. Moreover, lower recovery time and post-operative pain were reported in patients who underwent the endoscopic approach, compared to those who treated with the conventional repairing method (P<0.001).

**Conclusion:**

Endoscopic myringoplasty technique is a safe and effective way to improve hearing loss as much as the conventional method. However, due to the lower recovery time and post-operative pain, it seems to be the method of choice in myringoplasty surgery.

## Introduction

Chronic otitis media (COM) is a complex inflammatory and infective disorder that results in many healthcare visits across the world. This phenomenon is the main reason for hearing loss in different age groups (1). 

Middle ear inflammation with tympanic membrane perforation is the main feature of COM. The most common manifestations of COM include hearing loss, persistent otorrhea, tinnitus, and otalgia (2). 

Etiologically, COM has multifactorial origins, including immune response to microbial species, anatomical abnormal variations, and even genetic susceptibility (3,4). All of these factors lead to the inhibition of preventive pathways against the healing of the perforated tympanic membrane (5). 

Due to the wide spectrum of etiological factors and pathophysiological fundaments, the treatment approaches include a vast range of antimicrobial therapies to surgical interventions. 

Considering the role of bacterial infections in the creation and progression of COM, it has been shown that the isolation of both aerobic and anaerobic bacteria is expected in about 90% to 100% of the affected patients. Therefore, combination antibiotic therapy is the mainstay in the treatment of COM (6). However, the majority of patients with COM need surgical intervention to achieve the best outcome. Initially, microscopic tympanoplasty was introduced as a gold treatment approach in patients with COM. However, the visibility of different components of the middle ear may be potentially limited by employing this technique (7,8). Recently, the progression of an endoscopic, diagnostic, and therapeutic approach has received special attention as a new technique for the management of COM. In fact, the endoscopic approach can facilitate evaluation and access to a different part of the middle ear. Accordingly, it has been possible to diagnose any structural and pathological abnormalities as well as repair defects in different components of the middle ear. Few studies showed the superiority and feasibility of the endoscopic technique when comparing the benefits of microscopic and endoscopic treatment approaches for the management of the middle ear abnormality in patients with COM (9,10).It seems that the utilization of the endoscopic approach in the treatment of the patients with COM can effectively cover the potential limitations of interventions guided by microscopic approaches. However, insufficient data are available in therapeutic benefits and long-term outcomes of endoscopic versus conventional microscopic approaches for the treatment of COM. As a result, the present study aimed to compare the success rates and postoperative outcomes of endoscopic versus microscopic procedures in patients suffering from COM. 

## Materials and Methods

*Study Design*

This retrospective study was conducted on patients with chronic otitis media, who underwent cartilage myringoplasty at a tertiary referral center between 2014 and 2016 by one surgeon. 

*Study Population*

A total of 130 patients, who underwent unilateral cartilage myringoplasty were included in this study. A thorough history and physical examination were performed on all the subjects by an otologist. Moreover, all patients were clinically and radiologically evaluated, and no subject demonstrated evidence of cholesteatoma, inflammation, and otorrhea before the surgery. The Pure Tone Audiometry was conducted for each patient, and subjects with mild conductive hearing loss (pure-tone average < 40 dB HL) were selected for the study. This hearing threshold level was used as the baseline measurement. Regarding the exclusion criteria, the patients with the adhesive or atelectatic middle ear, and those who had cholesteatoma or granulation tissue, only hearing ear, revision surgery, and prolongation of surgery due to non-surgical causes were excluded from the study. 

*Surgical Procedures*

This study aimed to analyze the patient's medical records using one of the two therapeutic procedures, including transcanal endoscopic and postauricular microscopic myringoplasty.

**Fig 1:**
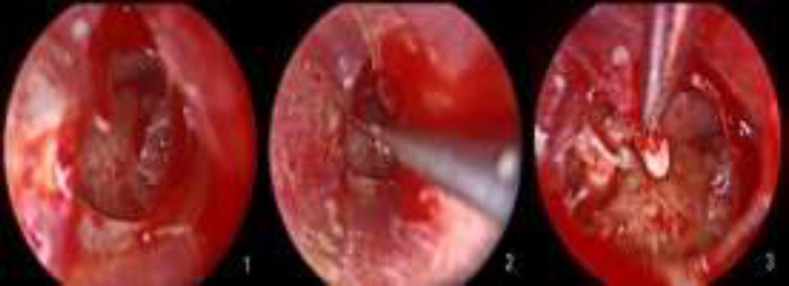
A. Limited perforation after freshening up the edges. B. After performing a radial incision and extending to the posterosuperior margin. C. Middle ear structures and ossicles are easily visualized by the endoscope

The transcanal endoscopic myringoplasty was performed under general anesthesia. The rigid endoscope (4.0-mm, 0°, 18-cm-long lens, HOPKINS^®^ telescope, Karl Storz Gmbh and Co.KG, Tuttlingen, Germany) was used for this purpose. Unlike the traditional methods, the tympanomeatal flap was not elevated, and the edges of the perforation were freshened up under the endoscopic guide. The middle ear mucosae and ossicles were checked by 0° and 30° endoscopes. In cases in which the size of the perforation limited the assessment of the middle ear, a radial incision was performed at the posterior-superior side of the tympanic membrane. This incision made it possible to assess the middle ear condition using a wide-angle telescope (Fig.1). 

The cartilage graft was harvested from the concha by preserving the perichondrium at one side. The thickness of the cartilage was reduced to 0.5 mm by the slicer. Subsequently, after placing the gel foam in the middle ear, the graft was placed medial to the edges of the perforation and annulus, and the perichondrium faced the lateral side (palisade cartilage myringoplasty underlay method). Only a thin layer of the gel foam was placed over the graft at the end. The microscopic cartilage myringoplasty was also performed under general anesthesia. After performing the postauricular incision, the tympanomeatal flap was elevated under the guide of the surgical microscope (Carl Zeiss OPMI microscope, Germany). After freshening the edges of the perforation and evaluation of the middle ear structures, the conchal cartilage was harvested, trimmed, and placed as discussed above using the underlay method (11). All the surgeries in each group were performed exclusively by an endoscope or microscope, and no change was made in the surgical method. In both groups, only nonsteroidal anti-inflammatory drugs (Ibuprofen 200-400 mg) were administered to all patients twice a day on the first-day post-surgery. 

*Data Collection*

The demographic characteristics and preoperative symptoms (i.e., otorrhea, otalgia, tinnitus, and vertigo) were determined using a retrospective review of the patients’ medical records in the hospital. The size of the perforation (i.e., small: < 25%, moderate: 25% to 75%, and large :> 75% of the surface of the tympanic membrane), site of the perforation (i.e., anterior, posterior, and central) and duration of anesthesia (from the induction to extubation) were assessed by evaluation of intraoperative video-recordings. Moreover, the postoperative pain was evaluated by visual analog scaling (VAS) method that rated the severity of the pain between 0 (for no pain) and 10 (for the worst pain imaginable) one day after surgery.The postoperative visit notes were assessed 1, 6, and 12 months after the surgery for any complication, presence of otorrhea, and surgical success which was defined as graft taking and absence of perforation (Table.1). 

**Table 1:** Baseline characteristics of the study groups

Item		Endoscopic group(n=75)	Microscopic group (n=55)	P-value
**Age (year)**		39.85 (18-68)	38.25 (16-77)	
**Gender, n (%)**	Male	23 (30.7%)	13 (23.6%)	0.724
	Female	52 (69.3%)	42 (76.4%)	
**Preoperative air bone gap (dB)** ^*^		25.2±6.2	24.9±17.3	0.665
**Last Otorrhea (months)**		7.97	11.02	0.097
**Operation side**	Right	37 (49.3%)	27 (49.1%)	0.988
	Left	38 (50.7%)	28 (50.9%)	
**Perforation location**	Anterior	3	10	0.449
	Posterior	31	13	
	Central	41	32	
**Clinical manifestations**				
**Hearing Loss**		74 (98.7%)	55 (100%)	0.957
**Otorrhea**		34 (45.3%)	41 (74.5%)	0.001
**Tinnitus**		5 (6.7%)	6 (10.9%)	0.531
**Otalgia**		1 (1.3%)	1 (1.8%)	0.999
**Vertigo**		N/A^**^	1 (1.8%)	0.998

*Average ABGs at 0.5,1,2,4 kHz. **Not Available.

Furthermore, the mean values of the air-bone gap (ABGs) were calculated at 0.5,1,2, and 4 kHz. 

The postoperative ABG minus baseline ABG was assessed to compare the hearing gain (Table.2). 

**Table 2:** Preoperative values of the air-bone gap (ABG) compared with 1, 6, and 12 months postoperatively.

	Pre-operative ABG [dB]	Postoperative ABG [dB]		
		1 month	6 months	12 months
**Endoscopic group**	25. 2±6.2	17.4±5.7	16.3±6.0	15.7±5.5
**Microscopic group**	24.9±7.5	19.7±6.4	16.6±6.2	14.4±5.4
**P-value**	0.809	0.0901	0.799	0.361

The data were assessed in SPSS software (version 16, SPSS Inc., Chicago, USA), and the results were presented as mean±SD for quantitative variables as well as the median and interquartile for categorical variables. In addition, the categorical variables were compared using the Chi-square test or Fisher's exact test, and t-test or Mann-Whitney U test were employed to compare the quantitative variables. A p-value less than 0.05 was considered statistically significant. 

*Ethical Considerations*

This anonymized chart review was conducted after obtaining ethical approval from the Ethics Committee of the relevant University of Medical Sciences (IR.IUMS.REC 1396.96-06-31-28397). It is worth mentioning that this study was carried out by tenets of the Declaration of Helsinki.

## Results

*Patients’ Characteristics*

Initially, 180 patients with COM and simple perforation of the tympanic membrane were candidates for cartilage myringoplasty with concha cartilage using the endoscopic or microscopic approach, which was administered randomly. A total of 50 patients were excluded from the study due to the presence of exclusion criteria, and finally, 130 patients (94 females and 36 males with a mean age of 39.18 years; age range:18-77 years) were included in the study and divided into endoscopic (n=75) and conventional microscopic groups (n=55).

Based on the patients’ statements, the last episode of otorrhea was 9.3±13.3 months before surgery (8.0±9.3 and 11.0±17.3 months for the endoscopic and microscopic groups, respectively). There was no difference between the groups regarding demographic characteristics, preoperative ABG values, pre-operative symptoms, and side of the surgery. Although the history of aural discharge showed a difference between the two groups, all patients were free of discharge for at least three months; however, it did not seem to be an important finding.

*Intra-operative Findings*

In total, small, moderate, and large perforation were revealed in 18.5%, 46.2%, and 35.4% of the patients, respectively. Regarding the site of perforation, 10.0%, 33.8%, and 56.2% of the patients suffered from anterior, posterior, and central perforation, respectively. Moreover, there was an anterior canal wall overhanging in five patients (two in the endoscopic group, and three in the microscopic group). In the microscopic group, all three patients needed drilling and canaloplasty; however, canaloplasty was not performed in the endoscopic group despite the overhang. Furthermore, the mean duration of anesthesia was significantly shorter in the endoscopic group, compared to the microscopic group (76.7±38.8 and 161.0±41.4 min, respectively, P< 0.001). There was a correlation between the size of the perforation and duration of anesthesia (94.8, 100.4, and 137.7 min for small, moderate, and large perforations, respectively, P<0.001). 

The size of the perforation correlates with the duration of anesthesia in the endoscopic group (45.0, 75.4, and 101.0 min for small, moderate, and large perforations, respectively, P<0.001); however, the perforation sizes were not significant in the second group (P>0.901). Nonetheless, the site of operation had no effects on the duration of anesthesia in both groups (P>0.293, and P>0.245 for the endoscopic and microscopic groups, respectively). 

*Postoperative findings*

The mean postoperative VAS scores were 2(1-2) and 4(3-5) in patients who underwent endoscopic and microscopic surgeries, respectively. Moreover, postoperative pain was significantly lower in patients who underwent endoscopic surgery (P<0.001). The success rate of operation was totally 96.9% with the failure of closing perforation in four cases. Additionally, the success rates of operation in endoscopic myringoplasty and microscopic surgery were determined at 97.3% and 96.4%, respectively, with no significant difference between the groups (P>0.05). 

Regarding the assessment of the relationship between operation success rate and baseline parameters (i.e., gender, age, the side of surgery, and size or site of perforation), there was no significant association between the groups in this regard. The granulation tissue and mild otorrhea at the site of the canal incision occurred in four patients in the microscopic group, which was controlled with topical medication and regular debridement. In the endoscopic group, no complications were present with the healing process. 

**Fig 2:**
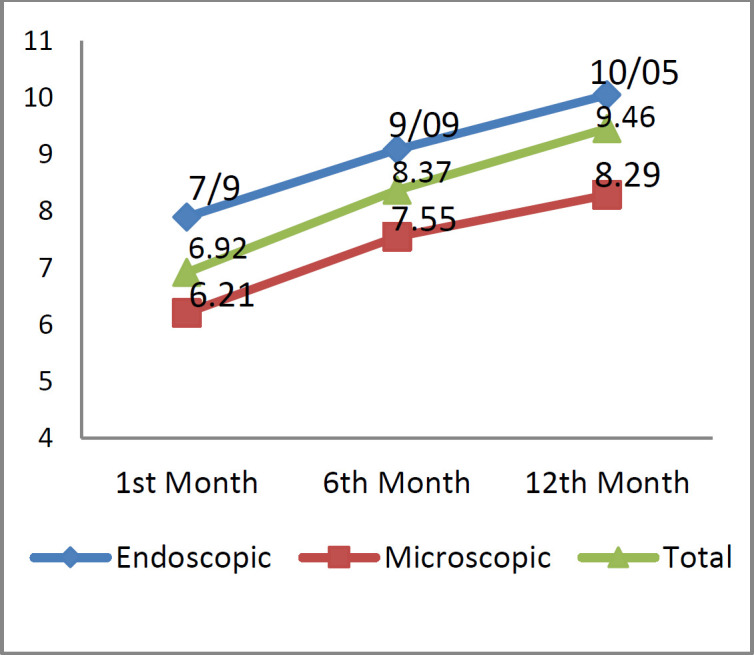
Postoperative hearing gain (dB) 1, 6, and 12 months after surgery

No significant difference was also reported between the two groups in terms of ABG values, as well as 1, 6, and 12 months postoperatively. Regarding the hearing gain, the obtained values were 6.92, 8.37, and 9.46 dB, 1, 6, and 12 months after surgery, respectively. The hearing gain was slightly higher in the patients who underwent endoscopic surgery (7.9, 9.09, and 10.05 dB, respectively), compared to those who scheduled for conventional microscopic approach (6.21, 7.55, and 8.29 dB, respectively); however, the difference was not statistically significant (P=0.063) (Fig.2). 

## Discussion

In a study conducted by Huang et al, in 2016, despite the similarity in postoperative perforations and equal improvements in hearing and ABG, the utilization of endoscopic technique led to less perioperative nausea or vomiting as well as a shorter operative time, compared to the microscopic approaches. Furthermore, they observed fewer tissue injuries and better cosmetic outcomes. Additionally, they suggested endoscopic myringoplasty as a better choice, compared to the conventional method (12). 

Similarly, Farahani et al. (2015) performed a study on patients over 15 years old and indicated that increased visibility of the middle ear structures (the epitympanic, posterior mesotympanic, and hypotympanic spaces) was the main advantage of endoscopic assessment, compared to the microscopic technique. Postoperative evaluation of the middle ear by endoscope revealed residual disease in four out of 13 patients after surgery. They emphasized using endoscope on searching hidden areas of the middle ear to prevent recurrences on specific pathologies (13). 

Daneshi et al. published the results of their study comparing two groups of patients, who underwent stapes surgery under a microscope and exclusively endoscopic approaches. They achieved similar hearing levels with shorter operating time and more patients’ satisfaction in the endoscopic group, compared to the traditional method (14).

In another study, Daneshi et al. demonstrated the feasibility and advantages of the endoscope in performing same-day bilateral tympanoplasty. Moreover, they achieved similar results after using the microscopic methods despite the ease of performing bilateral simultaneous surgery with an endoscope (15). 

In the same vein, Daneshi et al. has recently conducted a study managing class I and II glomus tympanicum tumors of the middle ear with an endoscopic approach. They were able to acquire improvement in the conductive hearing without any sensory neural hearing loss after surgery (16). 

In another study carried out by Ulku et al., the postoperative mean ABG was significantly higher in the endoscopic group, compared to the microscopic group (17). Lade et al. conducted a study on two groups of patients undergoing myringoplasty using endoscope and microscope. In the microscopic group, nine out of 30 patients required canaloplasty due to either external auditory canal overhang or ossicular assessment; nonetheless, in the endoscopic group, canaloplasty was not performed on any patient. 

Finally, they report similar graft take rate and audiometry results after 24 weeks of follow up. However, the lower rate of canaloplasty and better cosmetic outcome in the endoscopic group suggest endoscopic myringoplasty as an effective alternative for the conventional microscopic method with similar hearing benefits (18). In a retrospective study performed by Kuo et al., the comparison of the endoscopic and microscopic tympanoplasty demonstrated the feasibility of endoscope in tympanoplasty with the same benefit of hearing improvement and success rate with comparable complication rate. 

Moreover, the endoscopic group experienced much shorter operation time, smaller operation wounds, and lower medical costs (19). 

According to a retrospective study conducted by Choi et al., the surgical outcomes of type one tympanoplasty using two approaches were compared with each other. The patients were followed for three months, and they could show similar audiometric results, postoperative pain levels, and graft take rates. The study revealed shorter operation time and lower pain at the first postoperative day in the endoscopic tympanoplasty group, compared to the microscopic tympanoplasty group (20).

In a recent study carried out by Kaya et al. (2017), the feasibility of using the endoscope was shown to repair the tympanic membrane perforation in uncomplicated COM patients. In their endoscopic method, they did elevate limited tympano-meatal flap (described as limited to 1 to 6 o'clock and not extending to maleus) which led them to good hearing results and low complication rates (20). 

According to a currently conducted study, the superiority of the endoscopic approach over the conventional microscopic method was seen regarding lower postoperative pain severity and lower time for anesthesia. In the microscopic group, canaloplasty and drilling the anterior overhanging was mandatory in three cases, which was not necessary in two cases with an overhung canal in the endoscopic group. Furthermore, a slightly higher postoperative hearing gain was observed in the endoscopic group, which was not statistically significant. 

However, no reasonable explanation was found for this finding although the same method of grafting and graft materials was utilized in both groups. It seems that the transcanal approach without elevating tympano-meatal flap in the endoscopic group was responsible for less postoperative pain and faster recovery. As mentioned above, in four cases, granulation tissue and otorrhea were observed postoperatively in the microscopic group and none in the endoscopic group, which could be due to the canal incision. These mentioned findings could be the benefits of not elevating the tympano-meatal flap, which makes the procedure much easier, thereby accelerating the healing process.

A great advantage of using the endoscope was reported to assess the middle ear and ossicular chain before performing myringoplasty.

As described before, the utilization of a radial incision on the remnant of the tympanic membrane in small perforations facilitated better exposure to middle ear contents. 

Despite extending the incision, no complications were observed after using this method. However, endoscopic ear surgery has drawbacks, such as working with one hand, obscuring vision with minimum bleeding, and lack of three-dimensional views. The surgeon can overcome these disadvantages with experience over time. Most previous studies could obtain similar findings, compared to the outcomes of the current study. It seems that almost all superiorities of endoscopic myringoplasty compared to the conventional method refer to more visibility of middle ear parts by using the former tool. However, the results obtained in the present study and previous studies might be influenced by potential confounders, such as the experience of the surgeon. Regarding the limitations of the study, one can name the selection of the patients from one center, and non-randomized recruitment of the participants, as well as the unblinded nature of the review. These may increase the selection bias and the possibility of confounding the results. Therefore, it is suggested to use transcanal myringoplasty in these cases to decrease post-operation pain.

## Conclusion

In conclusion, there was no significant difference between endoscopic myringoplasty and microscopic surgery in this study. Moreover, similar operative success rates were reported regarding hearing gain. However, in addition to a higher ability to observe different components of the middle ear in the endoscopic approach, the application of this technique can lead to a more favorable outcome concerning a faster recovery to treat COM. Despite a small difference in the pain scores, it seems that endoscopic ear surgery is on average faster than the microscopic approach, which needs further investigation.
